# Peripartum Cardiomyopathy: An Intriguing Challenge. Case Report with Literature Review

**DOI:** 10.2174/157340309789317896

**Published:** 2009-11

**Authors:** Roberto Cemin, Rajesh Janardhanan, Massimo Daves

**Affiliations:** a Department of Cardiology, San Maurizio Regional Hospital, Bolzano, Italy; b Clinical Biochemical Laboratory, San Maurizio Regional Hospital, Bolzano, Italy; c Department of Cardiology, University of Virginia Health System, Charlottesville, Virginia, USA

**Keywords:** Peripartum cardiomyopathy, heart failure, echocardiography.

## Abstract

Peripartum cardiomyopathy is a relatively rare disease, which can have devasting consequences and should be promptly identified and correctly treated. Overall prognosis is good in majority of the cases, although some patients may progress to irreversible heart failure. Early diagnosis is important and effective treatment reduces mortality rates and increases the chance of complete recovery of ventricular systolic function.

We report of an interesting case with a favourable outcome and discuss about the clinical presentation, therapy and outcome of this condition.

## INTRODUCTION

Although the diagnostic criteria for peripartum cardiomyopathy (PCM) were established many decades ago, [1] its causes still remain unclear. The principal hypotheses with regards to the pathogenesis of this cardiomyopathy include an autoimmune response, an abnormal reaction to physiologic hormones or a viral aetiology [2-5]. PCM is a syndrome with symptoms of heart failure and signs of left ventricular systolic dysfunction, which manifest between the last month of pregnancy and the first 5 months postpartum [6]. Its incidence varies from 0.2% to 3% live births [6-8] and from region to region worldwide. The prognosis is generally good in the majority of cases although some patients progress to irreversible heart failure, heart transplantation or death [8, 9]. The diagnosis of PCM is made in the presence of symptoms and signs of heart failure strictly associated with partum and in the absence of other possible causes of dilated cardiomyopathy. The presence of ventricular systolic dysfunction is essential for diagnosis. Some echocardiographic parameters like an ejection fraction (EF) of less than 45% and an end-diastolic dimension index of greater than 2.7 cm/m^2^ have been proposed to better classify the dysfunction [10].

## CASE REPORT

A 34 years old lady was referred to our cardiological intensive care unit from a peripheral hospital. She had been admitted there for cough and dyspnoea which developed on the 15^th^ day after the delivery of her third child. A month prior to delivery she started feeling weak with dyspnoea and hypertension but declined treatment for hypertension and the dyspnoea symptoms were not investigated further. The chest x-ray on admission showed acute pulmonary oedema with bilateral pleural effusion and she was commenced on diuretics, ACE-inhibitors and antibiotics. Since the patient did not show any clinical improvement over the next 2 days, she was transferred to our intensive ward. On admission she was hypotensive (90 mmHg systolic blood pressure), with sinus tachycardia at 120 bpm with a gallop and diffuse rales on lung auscultation. ECG revealed diffuse T wave inversion. Echocardiography showed the presence of a severely impaired left ventricular systolic function with EF of 30%, fractional shortening of 11% along with severe mitral regurgitation and moderate tricuspidal regurgitation. Right and left ventricles appeared severely enlarged and diffusely hypokinetic. End-diastolic volume of the left ventricle was 118 ml/m^2^ and end-diastolic dimension 6.5 cm with an end-diastolic dimension index of 3.96 cm/m^2^. Biatrial enlargement was also noted. White cell count was 6700/cubic mm, Hb 9.8 g/dl, C reactive protein 0.21 mg/dl (normal value < 0.5), BNP 689 pg/ml (normal value < 100 pg/ml). The thyroid hormone profile was normal. Serological tests for the usual viral agents responsible for myocarditis were negative.

Antibiotic therapy was discontinued while the diuretics were continued. To correct the hypotension and improve perfusion, saline was infused gently (1.5 l/24 hours) while closely monitoring for any further evidence of fluid overload. ACE-inhibitors were cautiously initiated since the systolic blood pressure was low at 85-90 mmHg. Beta-blocker was started on the second day of admission at a low dose (bisoprolol 1.25 mg). The patient was also commenced on enoxaparin for thrombo-prophylaxis, because of the enlarged LV with significant dysfunction. 

Chest x-ray on day 15 showed resolution of the pulmonary oedema and pleural effusions. Follow up echocardiography showed improvement of mitral insufficiency, which became mild by day 30 along with normalisation of dimension and function of right ventricle and disappearance of tricuspid insufficiency. While the volumes of the left ventricle decreased, EF remained severely depressed (29% at discharge). BNP levels trended down to 614 pg/ml on day 15 after having risen to a peak level of 1111 pg/ml on day 5. The patient was discharged from the hospital on day 17 on furosemide 25 mg/day, lisinopril 20 mg/day, bisoprolol 2.5 mg/day, enoxaparin 8000 UI/day. Iron supplementation was also recommended.

During the follow up there was a continuous improvement of left ventricle dimensions and function and a decrease of BNP levels. Left ventricle EF increased to 40% by day 30 and BNP decreased to less than 100 pg/ml on day 45. The trend of left ventricle volumes and function is shown in Fig. (**1**) and that of BNP levels in Fig. (**2**).

## DISCUSSION

This case report highlights the diagnostic and therapeutic dilemma that physicians face when encountering patients with the rare, but interesting condition of peripartum cardiomyopathy which warrants prompt identification and treatment.

## CLINICAL FEATURES

PCM usually presents with classical symptoms and sign of systolic heart failure with ventricular enlargement and dysfunction seen on echocardiography. Often there is significant mitral and tricuspid regurgitation [11]. Unusual presentations include thromboembolism or hepatic failure secondary to heart failure. The development of heart failure and the usual time of diagnosis are during the post-partum period in more than 90% of the cases [12]. PCM can occur at any age with a higher incidence in women older than 30 years [1, 9].

The diagnosis of PCM is challenging because most women in the last months of a normal pregnancy or soon after the delivery experience dyspnoea, fatigue and pedal oedema. Symptoms and signs which should raise the suspicion of heart failure and could help the clinicians in the diagnosis, are the presence of paroxysmal nocturnal dyspnoea, nocturnal cough, new regurgitant murmurs, pulmonary crackles, jugular venous distention and hepatomegaly [13].

## PATHOGENESIS

The aetiology and pathogenesis seems to be multifactorial and poorly understood with the available literature rather conflicting. 

Gestational hypertension, tocolytic therapy and twin pregnancy have been proposed as possible risk factors because they were commonly associated with PCM [9]. These observations were not confirmed from other authors [14] who did not find any association between PCM and history of hypertension during pregnancy or use of tocolytc agents.

The association between PCM and twin pregnancy could support the theory of autoimmunity as a possible mechanism. This could depend on an excessive traffic of haematopoietic lineage cells from the foetus to the mother as manifest in twin pregnancy [15]. Usually the lower concentrations of these foreign proteins could contribute to tolerance of the foetus while increased levels could theoretically lead to the initiation of autoimmune disease [11]. The weak immunogenicity of the paternal haplotype of the chimeric cells or the naturally immunosuppressive state of the mother or both could avoid rejection of foetal cells during pregnancy. Following postpartum, the recovery of immune competence could trigger a pathologic autoimmune response against cardiac cells where haematopoietic cells have taken up residence during pregnancy and therefore myocardial cells are recognised as nonself [6].

Multiparity could be another risk factor for the development of PCM [13] and again, this observation has not been confirmed from other authors. In fact more than 50% of the patients are at their first or second pregnancy [9]. 

Molecular markers of an inflammatory process are found in most of the patients. 90% of the patients show high levels of plasma C-reactive protein that correlated positively with LV end-diastolic and end-systolic dimensions and inversely with LV ejection fractions [14]. This could indicate the chronic inflammatory state at baseline, which is higher in more unstable patients. The presence of a low-grade chronic inflammatory process could be due to the release of endotoxins and subsequent release of pro-inflammatory cytokines [16].

Interestingly, the possibility of developing PCM in a surrogate mother whose embryo was received from the commissioning couple of whom the mother was diagnosed with PCM following the delivery of her first child [17], points out the possible role of the presence of a transmissible agent, either infectious or non-infectious as well as genetic susceptibility. A hereditary predisposition is also suggested by familial reports of PCM [17] and strong consideration should be given to screening family members because PCM may be the forme fruste of a genetic predisposition to cardiomyopathy [6].

## PROGNOSIS

Overall prognosis of PCM is good in majority of the cases, although some patients may progress to irreversible heart failure. Progression of the condition requiring heart transplantation is described in 4% and death in 9% at a two years follow up [9]. Other studies showed a much higher mortality rate such as 15% or 32% at 6 months [14,18]. Patients who eventually die tend to have worse NYHA functional class, LVEF and larger LV dimensions at diagnosis [14]. There seems to be an initial high-risk period with 25-50% of the women dying within the first 3 months postpartum [19].

Sudden cardiac death has been reported to account for up to 50% of the mortality [20] and therefore attention should be paid to identify those patients who are likely to experience a late recovery of systolic function from those who should be considered for implantation of a cardioverter-defibrillator. Mortality rates have decreased over the past 10 years due to advances in medical therapy for heart failure and use of implantable defibrillators [21]. Normalisation of left ventricular systolic function occurs in 23% and in 54% of the patients respectively at six months [14] and at two years and is more likely if EF at diagnosis is more than 30% [9]. Higher left ventricular end diastolic dimension and lower fractional shortening at diagnosis seems to be associated to a worse prognosis. A fractional shortening of less than 20% and a left ventricular end diastolic dimension of 6 cm or greater was associated with a more than 3-fold higher risk for persistent left ventricular dysfunction [22]. 75% of the patients, who recover, have an EF of more than 45% at two months after diagnosis [23].

Complete recovery of systolic function occurs usually in the first 6 months after delivery [9] although the recovery phase need not be limited to the first 12 months. Continuing improvement was observed in the second and third year after diagnosis [7]. Persistence of the disease after 6 months portends worse survival [24].

To get an assessment of prognosis at the time of diagnosis, a dobutamine stress echocardiography study could be performed in non-critically ill patients. Inotropic contractile reserve accurately correlated with subsequent recovery of left ventricular function and usually associated with a benign prognosis [19]. MRI could be a useful alternative to dobutamine stress echocardiography in predicting outcome. Delayed gadolinium enhancement is more likely to be present in patients less responding to conventional therapy but could resolve over time in the recovering patients [25]. 

Even if left ventricular function recovers completely, exercise tolerance may remain abnormal and this could be more objectively assessed by an abnormal response to dobutamine stress echocardiography. 

## THERAPY

Management involves conventional therapy for heart failure with diuretics, ACE-inhibitors, beta-blockers and aldosterone antagonists. Angiotensin-receptor blockers should be added in case of ACE-inhibitors intolerance. Anticoagulant therapy should be considered in view of the low left ventricular EF, which predisposes to thrombus formation, especially in the peripartum period when a hypercoagulable state exists. In patients not improving on conventional therapy or in patients with critical hemodynamic state with cardiogenic shock, hemodynamic support with pressors should be considered. There have been some reports about the use of levosimendan [26] in non-responsive patients.

Non-responsive patients should be considered for heart transplantation even if there are some reports about effective use of extracorporeal membrane oxygenation [27], intraaortic balloon pump or mechanical assist devices.

Among patients who eventually recover, the withdrawal of heart failure medications was not associated with decompensation over a follow-up of 29 months [23]. The duration of heart failure treatment is determined by the patient's heart performance at rest and with exertion. Patients with normal EF at rest and during dobutamine could taper off medical therapy in 6-12 months; patients with normal EF at rest and abnormal EF during dobutamine should be treated for longer period with ACE-inhibitors and betablockers [28]. Patients who continue to have a depressed ventricular function at rest have a poorer prognosis and should receive medical therapy indefinitely. In any case, it seems reasonable to continue at least for a year with ACE-inibitors and betablockers even in case of complete recovery.

It is important to note that the use of ACE-inhibitors should be limited to after delivery since they have teratogenic effects. Other drugs like immunosoppressive drugs are still under evaluation.

## SUBSEQUENT PREGNANCIES

A subsequent pregnancy carries a high risk of relapse, significant decrease of left ventricular function and mortality. Mortality rate is described to be approximately 55% during subsequent pregnancy [29] even though it seems associated more with patients who entered the subsequent pregnancy with abnormal systolic function i.e. without making a complete recovery [30]. Complete recovery from a relapse is very rare. There is no consensus regarding recommendations for future pregnancy after PCM but patients whose left ventricular size or function does not return to normal should be counselled strongly to avoid subsequent pregnancy [6].

## CONCLUSION

PCM is a relatively rare disease, which can have devasting consequences and should be promptly identified and correctly treated. Early diagnosis is important and therefore women who develop symptoms of heart failure during pregnancy or shortly after should be investigated for this condition. Effective treatment reduces mortality rates and increases the chance of complete recovery of ventricular systolic function.

## Figures and Tables

**Fig. (1):**
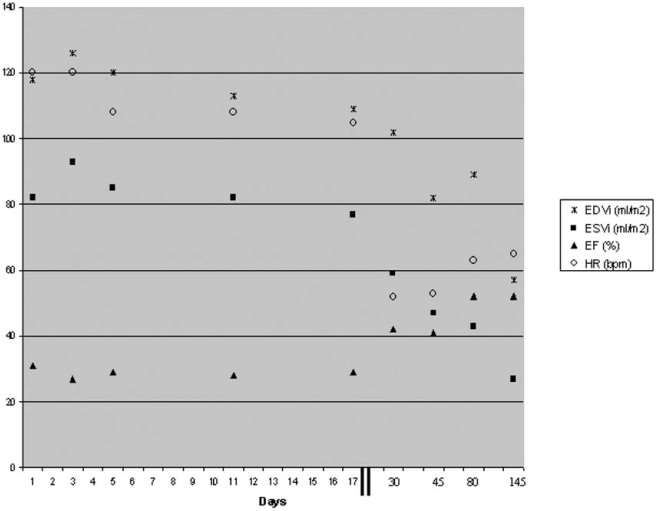
Trend of left ventricle volumes, function and heart rate. EDVi: end-diastolic volume index; ESVi: end-systolic volume index; EF: left ventricular ejection fraction; HR: heart rate.

**Fig. (2):**
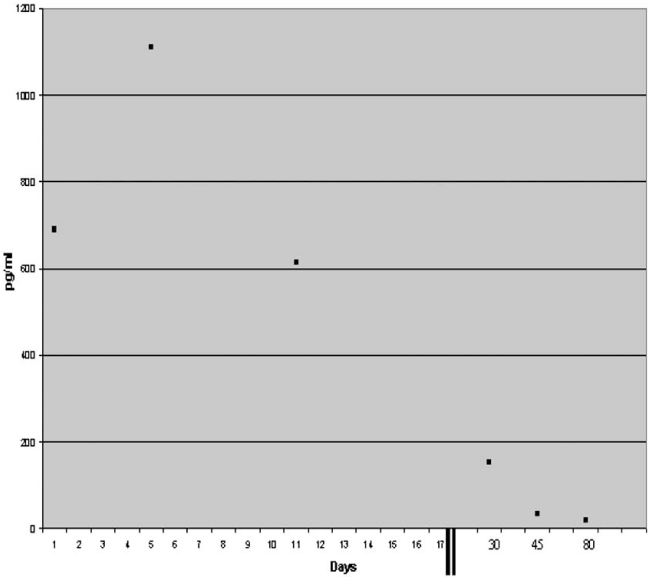
Trend of BNP levels over time.
